# A simplified high-throughput method for pyrethroid knock-down resistance (*kdr*) detection in *Anopheles gambiae*

**DOI:** 10.1186/1475-2875-4-16

**Published:** 2005-03-14

**Authors:** Amy Lynd, Hilary Ranson, P J McCall, Nadine P Randle, William C Black, Edward D Walker, Martin J Donnelly

**Affiliations:** 1 Vector Research Group, Liverpool Tropical School of Medicine, Pembroke Place, Liverpool, UK; 2 Department of Microbiology, Immunology and Pathology, Colorado State University, Fort Collins, Colorado, USA; 3 Department of Microbiology and Molecular Genetics, Michigan State University, East Lansing, Michigan, USA

## Abstract

**Background:**

A single base pair mutation in the sodium channel confers knock-down resistance to pyrethroids in many insect species. Its occurrence in *Anopheles *mosquitoes may have important implications for malaria vector control especially considering the current trend for large scale pyrethroid-treated bednet programmes. Screening *Anopheles gambiae *populations for the *kdr *mutation has become one of the mainstays of programmes that monitor the development of insecticide resistance. The screening is commonly performed using a multiplex Polymerase Chain Reaction (PCR) which, since it is reliant on a single nucleotide polymorphism, can be unreliable. Here we present a reliable and potentially high throughput method for screening *An. gambiae *for the *kdr *mutation.

**Methods:**

A Hot Ligation Oligonucleotide Assay (HOLA) was developed to detect both the East and West African *kdr *alleles in the homozygous and heterozygous states, and was optimized for use in low-tech developing world laboratories. Results from the HOLA were compared to results from the multiplex PCR for field and laboratory mosquito specimens to provide verification of the robustness and sensitivity of the technique.

**Results and Discussion:**

The HOLA assay, developed for detection of the *kdr *mutation, gives a bright blue colouration for a positive result whilst negative reactions remain colourless. The results are apparent within a few minutes of adding the final substrate and can be scored by eye. Heterozygotes are scored when a sample gives a positive reaction to the susceptible probe and the *kdr *probe. The technique uses only basic laboratory equipment and skills and can be carried out by anyone familiar with the Enzyme-linked immunosorbent assay (ELISA) technique. A comparison to the multiplex PCR method showed that the HOLA assay was more reliable, and scoring of the plates was less ambiguous.

**Conclusion:**

The method is capable of detecting both the East and West African *kdr *alleles in the homozygous and heterozygous states from fresh or dried material using several DNA extraction methods. It is more reliable than the traditional PCR method and may be more sensitive for the detection of heterozygotes. It is inexpensive, simple and relatively safe making it suitable for use in resource-poor countries.

## Background

The successful trials of pyrethroid insecticide-treated nets for malaria control in various endemic settings has led to the Roll Back Malaria initiative adopting the approach as one of the cornerstones of its malaria control programmes [1-3]. However, the increasing prevalence of insecticide resistance in *Anopheles gambiae*, the major vector of malaria in sub-Saharan Africa, threatens to compromise the successful use of insecticide-treated materials [4]. Resistance to pyrethroid insecticides was first seen in *An. gambiae sensu stricto *in West Africa [5] and has subsequently been detected in East Africa [6]. Whilst much of the observed resistance is thought to have been selected for by the use of pesticides in agriculture [7], there is already some evidence in East Africa that the introduction of treated bednets has selected for reduced susceptibility to permethrin [6].

One allele commonly associated with resistance to permethrin is the knock-down resistance or *kdr *allele. This allele encodes a modified voltage-gated sodium channel that has reduced sensitivity to DDT and pyrethroids. Molecular studies identified a single point mutation in the *kdr *allele that causes an amino acid substitution in domain II of the protein [8]. Two different mutations have been found in *An. gambiae*; the first causes a leucine to phenylalanine amino acid change and has been found in several West African countries [8-11], whilst the second found mainly in East African populations causes a leucine to serine substitution at the same amino acid position [6,12]. The importance of these mutations to the control of *Anopheles *mosquitoes is not yet fully understood. However, monitoring its frequency, as a rapid indicator of the development of resistance, should be an integral component of insecticide resistance management programmes.

The most commonly used method for identifying the *kdr *mutations involves a multiplexed PCR technique. Single Nucleotide Polymorphism (SNP) detection is problematic with simple PCR approaches, requiring the use of highly toxic reagents [13] or prohibitively expensive equipment. Many of these approaches are difficult to transfer to field laboratories where the ability to monitor gene frequencies is most acutely needed. The technique detailed here, adapted from one originally designed by W.C. Black IV requires only a thermal cycler and provides an easily interpretable, colorimetric genotyping system. No toxic reagents are involved. While this system has been specifically designed to assay *kdr *resistance allele frequencies in *An. gambiae*, it is broadly applicable where target-site insensitivity is an important mechanism of resistance to insecticides and to chemotherapeutics.

## Methods

### Mosquito strains and bioassays

Specimens were obtained from laboratory colonies of RSP (a homozygous line for the East African *kdr *mutation), Kisumu (a susceptible line from Kenya, established in 1953), and Odumasi (a partially resistant line, not yet fixed for the West African *kdr *mutation). Adult females were stored at -20°C before extraction. Field caught specimens were collected using resting catches from Asembo in western Kenya in May 2004, and by pyrethrum spray collections in Odumasi, Ghana in June 2003. Samples were dried over silica gel for later analysis.

### PCR

All PCR reactions were performed in ABI GeneAmp^® ^PCR system 2700 or MJ Research PTC-200 DNA Engine thermal cyclers. Primers Agd1 and Agd2 [8] were used to amplify a 293 bp fragment from domain II of the voltage-sensitive sodium channel protein sequence (EMBL #Y13592). PCR was carried out with the DNA of 1/80^th ^or 1/160^th ^of a single mosquito in a 25 μl volume with a final concentration of 1x Buffer, 2.0 mM MgCl_2_, 0.2 mM dNTP's (Sigma dNTP-100), 0.3 μM each primer (Qiagen), Taq DNA polymerase 0.034 U/μl (Qiagen 201203). Reaction conditions were 94°C for 4 min, 25 cycles of 94°C for 25 sec, 56°C for 20 sec, 72°C for 8 sec; and a final extension step of 72°C for 10 min (modified from [12]). Artificial heterozygote controls were created using DNA from two homozygous samples.

DNA from a single mosquito was extracted using the Livak method, [14] or the Ballinger Crabtree method [15] and resuspended in 100 μl or 200 μl of ddH_2_0.

Species identification was carried out on all specimens using a PCR method [16]and specimens were characterized for *kdr *status using PCR methods [8,12]. PCR products were visualized under UV light on 1.5% agarose, 0.5x TBE gels stained with ethidium bromide.

### Hot Ligation

3 μl of PCR product from the above reaction was used in a hot ligation with Detector and Reporter oligonucleotides (MWG Biotech) (Table 1). Aliquots were made for each oligo pair containing 1 μM detector and 1 μM reporter in ddH_2_0. A 20 μl reaction volume containing 1x Buffer, 50 nM detector and reporter mix and 0.05 U/μl Ampligase^® ^(Cambio A32250) was set up for each oligo pair. Four reactions were set up for each PCR sample to test for the East and West resistant alleles and the susceptible allele (two different oligo pairs must be used to test for the susceptible allele in these assays, as the potential oligo binding site differs by one base pair). The reaction conditions were 95°C for 5 min, 25 cycles of 94°C for 1 min, 58°C for West African *kdr *detection or 60°C for East African *kdr *detection for 2 min; with a final hold at 4°C. Ligated products were kept at 4°C in the dark and used as soon as possible for SNP analysis.

**Table 1:** Oligonucleotide sequences used in the Hot Ligation

**Description**	**Oligo Name**	**bp Position^a^**	**Oligo sequence 5' – 3'**	**Modifications**
Suspt. East *kdr *detector	Kdr104L-DTe	311-15*i*	ATTTGCATTACTTACGACTA	5' Biotin
Resist. East *kdr *detector	Kdr104S-DTe	311-15*i*	ATTTGCATTACTTACGACTG	5' Biotin
East *kdr *reporter	Kdr104-RTe	291–310	AATTTCCTATCACTACAGTG	5' Phosphorylation 3' Fluorescein
Suspt. West *kdr *detector	Kdr104L-DTw	312-16*i*	AATTTGCATTACTTACGACT	5' Biotin
Resist. West *kdr *detector	Kdr104F-DTw	312-16*i*	AATTTGCATTACTTACGACA	5' Biotin
West *kdr *reporter	Kdr104-RTw	292–311	AAATTTCCTATCACTACAGT	5' Phosphorylation 3' Fluorescein

^a^Using sequence from Martinez-Torres *et al*., as reference; *i *intron 2 position.

### SNP Detection

96-well plates (VWR 402 200 402) were prepared using 100 μl of 5 μg/ml streptavidin (Sigma S4762) per well. The plate was left to dry overnight and then washed 4 times in 250 μl of 1 x PBS with 0.1% v/v Tween 20. Buffer was removed by tapping the plate upside down and 200 μl of blocking solution (1x PBS, 0.1%v/v Tween 20, 2%w/v BSA) added for 1 hour. Four more washes of 250 μl with PBS were carried out before plates were covered with a plastic seal and stored at 4°C for up to one week.

20 μl of TNE (10 mM Tris-HCl pH7.5, 1 mM EDTA pH 8.0, 0.2 M NaCl) was added to the hot ligation reaction and then all 40 μl was placed in a well of the streptavidin plate and allowed to incubate at room temperature for 30 min in the dark. The ligation reaction was carefully removed with a multichannel pipette and the plate washed twice in 250 μl of freshly prepared wash buffer 1 (10 mM NaOH, 0.05%v/v Tween 20) and then twice in 250 μl of wash buffer 2 (0.1 M Tris-HCl pH7.5, 0.15 M NaCl, 0.05%v/v Tween 20).

40 μl of 75 mU/ml HSP-conjugated antifluorescein Ab (Roche 1 426 346) solution in 1% w/v BSA solution was placed in each well and incubated at room temperature for 30 min. The plate was then washed three times in 250 μl of wash buffer 2. All buffer traces were removed by tapping the plate upside down on a paper towel and 100 μl of room-temperature TMB solution (Roche BM Blue Pod Substrate 1 484 281) added. At least 5 min were allowed for the colour to develop before plates were scored. Plates were read at 680 nm in a Molecular Devices Versa Max plate reader to provide a quantitative method of scoring which could be compared to the visual method of scoring to check reliability.

## Results and Discussion

**Figure 1:**
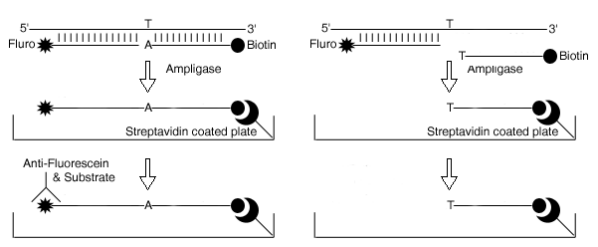
Schematic of Hot Oligonucleotide Ligation Assay for West African Allele

**Figure 2:**
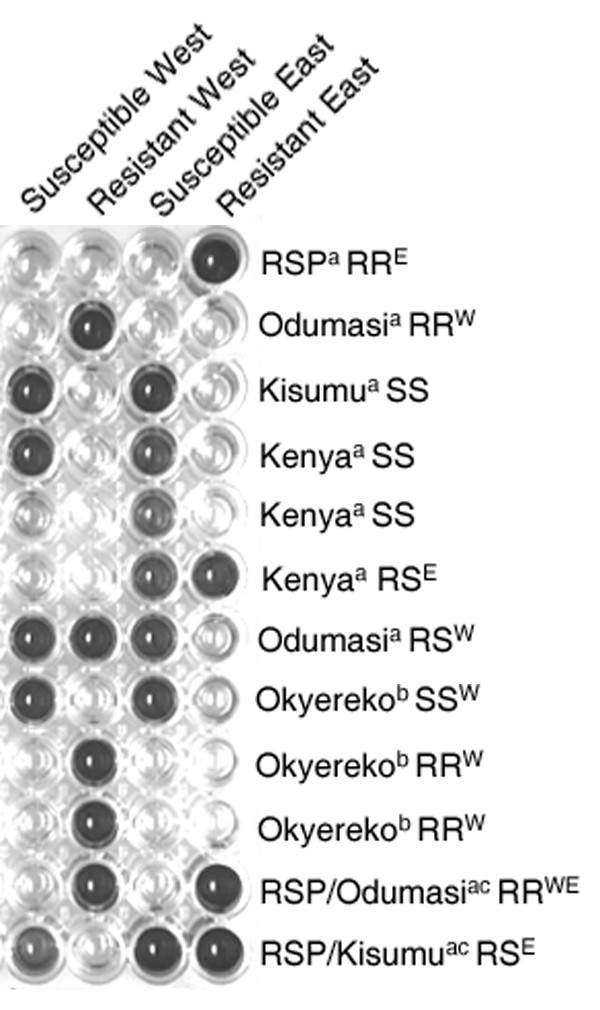
**Photograph of HOLA plate, including DNA extraction method and expected results. **Abbreviations: SS, homozygous susceptible. RR, homozygous resistant. RS, heterozygous. ^a^Livak [14] extraction method ^b^Ballinger-Crabtree [15] extraction method ^c^Artificially created heterozygote

A schematic of the HOLA approach is given in Figure 1 and a photograph of the HOLA 96-well plate is shown in Figure 2. Susceptible individuals score positively for both the East and West African susceptibility tests although a somewhat weaker reaction may be seen in East African susceptible individuals for the West Susceptibility test. Resistant individuals show a positive colour change only for their specific *kdr *allele. Heterozygotes are easily distinguishable. The protocol presented here for *kdr *detection is reliable and gives unambiguous results (Table 1). Visual and colorimetric scoring results were always comparable (data not shown). A double-blind trial was carried out on 12 wild-caught specimens of *An. gambiae *from East Africa compared to the commonly used PCR multiplex approach. The genotype was unambiguously determined by the HOLA technique, whereas the PCR results were more difficult to interpret and often required a repeat reaction (Table 2). There was one discrepancy between the two approaches which was not resolved after repeated analyses (Specimen Kenya 3, Table 2). It is believed that the HOLA method gave the correct result since three HOLA repetitions were carried out on the sample which all scored the specimen as heterozygous. Contamination may be excluded as a cause of this discrepancy as HOLA reactions were performed before and after the PCR tests. Furthermore the *kdr *allele is rare in the Kenyan population [17] and so would be much more likely to occur more frequently in a heterozygous rather than homozygous state.

**Table 2:** Double blind trial of HOLA approach versus conventional PCR

**Specimen**	**HOLA**	**PCR 1^e^**	**PCR 2^e^**
NK5^a^	SS	SS	SS
NK6	SS	X	SS
NK7	RR	RR	RR
NK8	SS	SS	SS
Kenya 1^b^	SS	X	SS
Kenya 2	SS	X	SS
Kenya 3	RS	X	RR
Thyolo 7^c^	SS	SS	SS
Thyolo 33	SS	SS	SS
Thyolo 34	SS	X	SS
Thyolo 64	SS	X	SS
Thyolo 75	SS	X	SS
RSP^d^	RR	RR	RR

^a^Specimens labelled NK collected by Pie Muller, Ben Oloo, and Nadine Randle from Asembo, Kenya on 05/2004, DNA extracted by Ballinger-Crabtree method [15] on 09/2004. ^b^Specimens labelled Kenya collected by Pie Muller, Ben Oloo, and Nadine Randle from Asembo Bay, Kenya on 05/2004 DNA extracted by Livak method [14] on 08/2004. ^c^Specimens labelled Thyolo collected by Philimon Tambala and Bill Hawley from Thyolo, Malawi on 01/1995, DNA extracted by Ballinger-Crabtree method [15] on 09/1997. ^d^Specimen from RSP colony. Abbreviations: SS, homozygous susceptible. RR, homozygous resistant. RS, heterozygous. ^e ^Conditions for the PCR reactions were identical.

The HOLA method allows for over 40 samples to be screened on a single microtitre plate. As shown in Figure 2, the method works for a variety of DNA extraction techniques, on fresh and stored material. Although costs per reaction are slightly higher than for the traditional multiplex PCR, the greater reliability ensures that repeat reactions are unlikely to be required, reducing costs in the long term. In addition, since this technique dispenses with the need for gel electrophoresis apparatus there is a lower initial equipment outlay, greater comparative safety and greater ease of this technique, making the method ideal for field laboratories.

## Conclusion

The HOLA method allows fresh and stored *An. gambiae *mosquitoes to be characterized for the East and West African *kdr *mutations. Homozygotes and heterozygotes can be easily distinguished using low cost equipment and a simple methodology which makes this technique suitable for use in resource-poor countries. In our hands the method is more reliable than the current multiplex PCR approach, less ambiguous and may be more sensitive for the detection of heterozygotes.

## List of Abbreviations used

DNA – Deoxyribonucleic acid.

ELISA – Enzyme-linked immunosorbent assay

HOLA – Heated oligonucleotide ligation assay.

*Kdr *– Knock down resistance.

PCR – Polymerase chain reaction.

SNP – Single nucleotide polymorphism.

## Authors' contributions

AL developed the HOLA method for the *kdr *mutation and drafted the manuscript. HR conceived of the study and participated in its design. NPR carried out the multiplex PCR. PJM and EDW helped draft the manuscript. WCB developed the HOLA technique. MJD participated in the design of the study and substantially helped draft the manuscript.
